# Preparation and Investigation of Intelligent Polymeric Nanocapsule for Enhanced Oil Recovery

**DOI:** 10.3390/ma12071093

**Published:** 2019-04-02

**Authors:** Fang Shi, Jingchun Wu, Bo Zhao

**Affiliations:** 1 Key Laboratory for EOR Technology (Ministry of Education), Northeast Petroleum University, Xuefu Road 99, Daqing 163318, China; 2 Daqing Oil Field Co., Ltd. No. 6 Oil Production Plant Test Brigade, Daqing 163318, China; shifang0502@126.com

**Keywords:** oil displacement agent, amphiphilic Janus nanoparticles, emulsifying resistance coefficient, intelligent polymeric nanocapsule, enhanced oil recovery

## Abstract

Micro-/nanomotors colloidal particles have attracted increasing interest as composite surfactants, owing to the combined advantages of both Janus solid surfactants and micro-/nanomotors. Here we put micro-/nanomotors colloidal particles into hollow polymeric micro-encapsulates. An intelligent polymeric nanocapsule was prepared for enhanced oil recovery by the self-assembly method. The particle size range of the polymeric capsule can be controlled between 20 to 1000 nm by adjusting the cross-linking thickness of the capsule’s outer membrane. The average particle size of polymeric capsules prepared in the study was 300 nm. The structure and properties of the Intelligent polymeric nanocapsule was characterized by a wide range of technics such as Fourier transform infrared spectroscopy, scanning electron microscopy by laser diffraction, fluorescence microscopy, pendant drop tensiometer, laser particle size instrument, and interface tension analyzer. It was found that the intelligent polymeric nanocapsule exhibited significant interfacial activity at the oil-water interface. When the Janus particles’ concentration reached saturation concentration, the adsorption of the amphiphilic nanoparticles at the interface was saturated, and the equilibrium surface tension dropped to around 31 mN/m. When the particles’ concentration reached a critical concentration of aggregation, the Gibbs stability criterion was fulfilled. The intelligent polymeric nanocapsule system has a better plugging and enhanced oil recovery capacity. The results obtained provide fundamental insights into the understanding of the assembly behavior and emulsifying properties of the intelligent polymeric nanocapsule, and further demonstrate the future potential of the intelligent polymeric nanocapsule used as colloid surfactants for enhanced oil recovery applications.

## 1. Introduction

The chemical flooding agents are of great practical interest because of their extensive application in petroleum exploitation [1]. The application of chemical flooding agents in oil recovery face some technical and economic challenges, such as surfactant adsorption on rocks caused by increased dosage [2], the large size of polymer molecular coils, and their high cost [3], as well as the percolation properties [4]. The disorderly nonlinear movement of chemical flooding agents in reservoirs makes it difficult to strip the remaining oil efficiently without chromatographic separation between chemical agents [5]. Intelligent micro-and polymeric nano-capsules for oil displacement have been designed to optimize the structure of the composite chemical flooding agent. 

Polymeric nanocapsules replace traditional surfactants by nano-active particles, and polymer films replace traditional polymer for oil displacement. Composite chemical flooding agents are formed by assembly. The intelligent oil displacement agent is a promising candidate for improving oil and gas recovery.

Intelligent nano-capsule polymer is a new form of binary composite chemical flooding agent, which reforms the structure of the composite displacement agent. The capsules are similar to pharmaceutical drugs carriers. The intracapsular agent is an asymmetric surfactant nanoparticle (referred to as Janus Particles (JPs)) [6]. The capsule shell is a nano polymer film, which encapsulates these JPs in the capsule to isolate the JPs from the outside environment. Compared with conventional low molecular weight scale surfactants, JPs are characterized by small size, strong emulsification, high stability and low adsorption.

The capsule shell is decomposed into polymeric dispersion over time, which can suspend JPs efficiently. When the JPs are released to the oil-water interface, with the fluid migration, the particles form a close accumulation at the oil-water interface, and gradually form a bridge. With the subsequent fluids scouring, the particles are divided into two paths for migration. Some particles are bonded to this bridge temporarily to plug high permeability channels, and some particles are moved to high oil-bearing areas and crude oil emulsification. The emulsified liquid is transported to the vicinity of the high permeability channel in the following part of the fluid, while the original plugging channel breaks through with the continuous erosion pressure of the other part of the particle, and the fluid is transported to the bottom of the oil well. The intelligence of the polymeric nanocapsule is to selectively identify the target oil by assembling or covalently coupling groups with specific recognition function to the surface of microcapsules. Intelligent nano-displacement technology enables the displacement agent to reach target location of the reservoir, and is intelligent enough to selectively and capture crude oil in the reservoir, enlarge the sweep volume dynamically along the way, and to improve the efficiency of washing the oil.

At present, there are two main types of nano displacement agents for enhancing oil recovery in low permeability reservoirs [7,8]. One type of oil displacement agent is mainly based on the optimization of surfactants, such as uniformly modified nanoparticles and Molecular Deposition (MD) films. The other is based on the optimization of polymers, such as modified nano copolymers and gels [9,10]. MD film flooding agent [9] uses aqueous solution as transfer a medium, and self-assembles monolayer MD super film on different interfaces of the reservoir system by a strong electrostatic interaction between ions. Large heat is released, and an "energy field" is formed to improve oil recovery after film formation. The target displacement agent combines the advantages of the above technologies and displacement mechanism to form an assembly of asymmetric nanoparticles and nanofilms. Modified nanoparticles can reduce the capillary resistance of reservoirs, then reduce pressure and increase injection, and expand the sweep volume microscopically, which provides technical guidance for the scientific problems of “difficult injection and difficult production” in low permeability reservoirs [11]. Hydrophilic modified nanoparticles can reduce capillary resistance to the greatest extent [12]. Hydrophobic modified nanoparticles can be used in rock while reducing pressure and increasing injection [13]. Super hydrophobic film is formed on the stone surface, and the residual oil on the rock surface is peeled off. Asymmetric amphiphilic nanoparticles endow amphiphilic nanoparticles with amphiphilic properties, optimize the injection effect and improve oil and gas recovery. Compared with low molecular weight surfactant and homogeneous particle stabilized emulsion, two nanoparticles with high affinity will be tightly arranged at the oil-water interface in combination with their heterogenous and strong emulsifying properties [14,15]. The two tight particles as a solid surfactant can effectively stabilize the emulsion and obtain long-term stability. However, nano-film is used to avoid wasting the resources of amphiphilic nanoparticles near the wellbore. It can assemble or covalently couple groups with a specific recognition function on the surface of the nano-film, so that nanocapsules can interfere or adjust their own migration trajectories in the reservoir environment, and finish where they are most needed. It aims to transport chemicals to achieve the goal of intelligent capsule drive. Nevertheless, smart polymeric nanocapsules have not been applied in any oil field to improve oil and gas recovery technology.

In this article, asymmetric amphiphilic nanoparticles, Janus particles (JPs for short), have been developed by covalent coupling modification. JPs provide an asymmetry that makes a single particle have new, different physical and chemical properties, especially the directionality of particles. Amphiphilic nanoparticles have high specific surface area, high thermal stability and the effect of reducing capillary resistance [16]. They can reduce pressure and increase injection, reduce oil-water interfacial tension to ultra-low, enhance the ability of emulsifying crude oil [17], overcome the disorder migration of Brownian motion, and realize directional movement [18]. The capsule wall is endowed with responsive special groups to identify reservoir oil saturation [19]. In the fluid migration process, the migration route is adjusted to release nanoparticles in the most suitable location. Additionally, the plugging capabilities of five oil displacement systems were systematically studied, including traditional surfactants, uniformly modified nanoparticles, asymmetrically modified nanoparticles, uniformly modified nanoparticles microcapsules, and asymmetrically amphiphilic nanoparticles microcapsules.

## 2. Materials and Methods

### 2.1. Materials

Nano-SiO_2_ particles (30 nanometers in diameter) were purchased from Shanghai Keyan Industrial Co., Ltd. (Shanghai, China). Fully refined paraffin particles (melting temperature range: 55 °C to 58 °C) were purchased from Beijing Haibeisi Company (Beijing, China). Gamma-methacryloxypropyl trimethoxysilane (gamma-MPS) (99%) was purchased from Dongguan Shanyi Plasticizing Co., Ltd. (Dongguan, China), acetone and cetyltrimethylammonium bromide (CTAB) from Tianjin Photosynthetic Fine Chemical Research Institute (Tianjin, China), fluorescein isothiocyanate (FITC), Phosphate buffer solution (PBS) and dimethyl sulfoxide (DMSO) were purchased from Sigma Aldrich (Saint Louis, MO, USA).

### 2.2. Preparation of Asymmetric Amphiphilic Nanoparticles

A double- affinity asymmetric Janus colloid nanoparticles were synthesized by the Pickering emulsion change assisted method [20,21,22]. The nanoparticles were divided into two parts that were separated in two phases, oil and water, respectively. In order to avoid the formation of Janus colloid by the free rotation of nanoparticles, the nano silica particles were embedded in solid paraffin. In the solid paraffin/water two-phase emulsion interface, nano-silica particles were selectively modified with silane coupling agent to give them selectivity.

**Figure 1:**
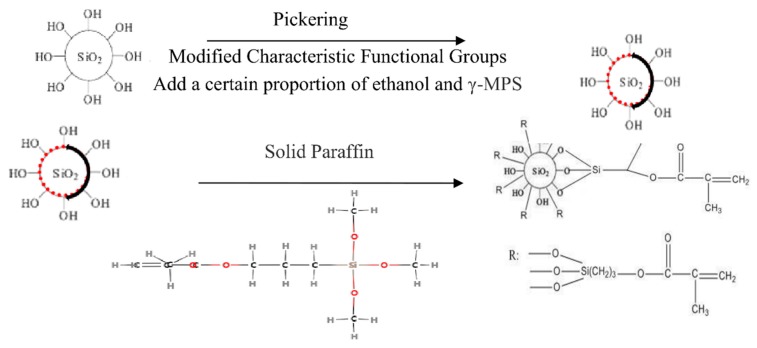

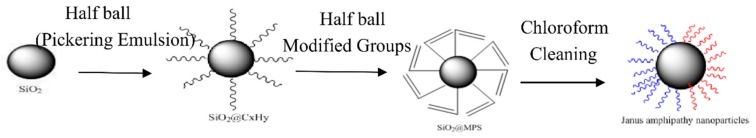
Synthesis scheme of asymmetric amphiphilic nanoparticles.

The synthesis scheme of asymmetric amphiphilic nanoparticles is shown in Figure 1, 0.5 g Nano-SiO_2_ particles and 3 g solid paraffin were dispersed in 20 g deionized water and emulsified for 1 h in a high-speed mixer (Chengdu Saikolong Machinery Equipment Co., Ltd., Sichuan, Chengdu, China) at 2800 r/min. Immediately after the emulsification, the emulsion was cooled in the refrigerator freezer to solidify the paraffin wax. The surface of solidified paraffin emulsion droplets was washed with deionized water to remove the non-adsorbed Nano-SiO_2_ particles, and the solidified paraffin emulsion droplets were vacuum dried at 45 °C. In the typical silylation process, dried paraffin emulsion droplets were dispersed in 0.1% gamma-MPS ethanol solution and reacted at room temperature for 72 h. After the reaction, the paraffin droplets were filtered out, and the surface of the droplets was washed with ethanol to remove unreacted gamma-MPS and unabsorbed Nano-SiO_2_ particles. Then the paraffin was dissolved with trichloromethane, and the modified Nano-SiO_2_ particles were collected by centrifugal rinsing, and then dried in a vacuum drying box (German Binder Company, Tutlingen City, Baden-Wutenberg State, Germany) for 24 h and after this, characterized.

### 2.3. Preparation of Fluorescent Amphiphilic Nanoparticles

**Figure 2:**
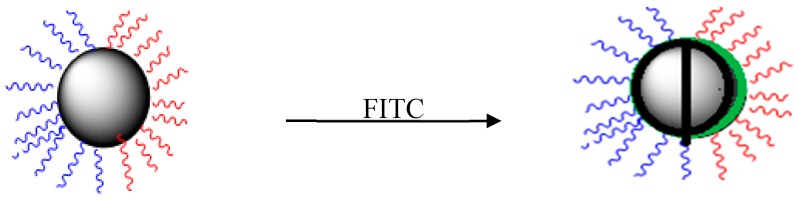
Fluorescence labeling schematic.

FITC (10 mg) and PBS (20 mL, pH 7.5) were added to 1% acetone solution, stirred sufficiently [23], and then the prepared amphiphilic nanoparticles Janus (20 mg) were added to the mixed system. JANUS particles were separated by centrifugation after labeling with FITC in a dark environment, and stirring at room temperature for 12 h. PBS was washed three times to remove the unreacted fluorescent agent, and then FITC-labeled Janus particles were suspended in DMSO for fluorescence microscopic observation. The preparation diagram is shown Figure 2.

### 2.4. Interface Properties of Amphiphilic Nanoparticles 

#### 2.4.1. Types of Surfactants 

The ability of surfactants to reduce surface/interfacial tension depends mainly on the tightness of surfactant molecules in the adsorption layer on the solvent surface [24]. According to the research on the properties of common surfactants used in oil fields, it is found that [25] nonionic surfactants with the same hydrophobic chain length have a stronger ability to reduce surface/interfacial tension than anionic surfactants, due to the charge of anionic surfactants and reservoir rock repulsion. 

#### 2.4.2. Surfactant Hydrophobic Group 

The types, lengths and structures of hydrophobic groups affect the ability of surfactants to reduce surface tension [26,27]. The surface/interfacial tension of fluorocarbon chain surfactants and silicone surfactants is much lower than that of hydrocarbon chain surfactants with carbon, and the difference is 1–2 times under the same conditions [28,29]. The ability of reducing surface/interfacial tension increases slightly with the increase of hydrophobic group chain length. In the absence of precipitation, hydrophilicity increases, interfacial adsorption also increases, and the surface/interfacial tension decreases. 

#### 2.4.3. Effect of Salt Water on Surface Tension of Surfactants 

Inorganic salts have a significant effect on the surface surfactant system of ionic surfactants [30]. Salt tolerance of surfactants depends on the structure of surfactants. Janus nanoparticles contain an Acryloxy Group structure, which enhances the affinity between solid surfactants and water molecules.

The addition of inorganic salts weakens the electric repulsion between the adsorbed layer and the surface of surfactant ions in micelles, makes them more closely arranged, and enhances the ability to reduce the surface/interfacial tension [31]. For ionic surfactants, such as inorganic cations, one mole of calcium ion binds to two moles of surfactant anions, which greatly enhances the tightness of the membrane arrangement, and greatly reduces the interfacial tension. For the non-ionic type, there is no double layer structure. 

#### 2.4.4. Effect of Temperature on Surface Tension of Surfactants 

Temperature has little effect on ionic surfactants, while for non-ionic surfactants, the hydrogen bond of polar head is easily destroyed at high temperature [31], which increases the hydrophobicity of surfactants and decreases the surface/interfacial tension. 

In conclusion, the surface/interfacial tension of surfactants mainly depends upon two factors: 1. The structure of the surface adsorption layer (the state and tightness of the total hydrophobic chain arrangement of the surface adsorption layer); 2. the properties of hydrophobic groups (weak interaction, weak surface/interfacial tension). Therefore, it is important to evaluate the interfacial properties of different systems of intelligent flooding agent for matching the adaptability of reservoir environment.

#### 2.4.5. Emulsification Mechanism of Asymmetric Amphiphilic Nanoparticles

**Figure 3:**
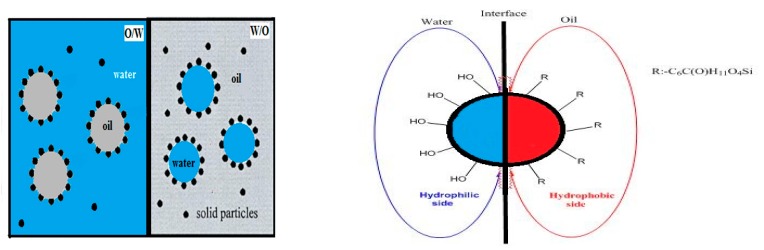
Schematic diagram of the Pickering emulsification stabilization mechanism of homogeneous Janus nanoparticles and asymmetric amphiphilic Janus nanoparticles.

At present, the emulsifying stabilization mechanism of the Pickering emulsion method, which is relatively well-known, is that the solid particles form a compact interfacial film on the oil–water interface, thus forming a stable emulsion [32,33]. Compared with the traditional surfactant, the film formed on the oil–water interface of the Pickering emulsion is a solid ion membrane rather than a molecular aggregate. The schematic diagram is shown in Figure 3. This property makes the physical and chemical properties of Pickering emulsion more stable, and is not affected by shear stress.

### 2.5. Characterization of the Modified Nanoparticles

The surface morphology of the modified Janus colloid was observed by scanning electron microscopy (SEM, JEOL Co., Tokyo, Japan). The chemical modification of SiO_2_ particles was detected by a WQF-410 Fourier Transform Infrared Spectrometer (FTIR) of Malvern Company, Massachusetts, UK. The surface morphology of paraffin emulsion droplets was characterized by field emission scanning electron microscopy (FEI Sirion 200, JEOL Co., Tokyo, Japan) from the Netherlands. The surface activity of Janus particles was measured by the Tracker interfacial dilatation/compression rheometer made by Teclis, Odom Company, Arras, France. The particle size distribution of paraffin droplets in paraffin/water emulsions was measured by the Mastersizer 3000 laser particle size analyzer (Malvern Instruments Ltd., Malvern, UK). The fluorescence characteristics of the prepared fluorescent Janus particles were characterized by FV1000 laser confocal microscopy of Olympus Company, Tokyo, Japan.

### 2.6. Assembly of Smart Microcapsules

**Figure 4:**
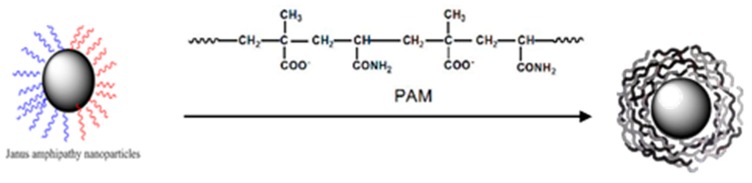
Self-assembly process of microcapsules and nanocapsules.

Based on the characteristics of core-shell structure, nano-material A is encapsulated in another nano-material B by a certain force to form a micro-nano-scale orderly assembly structure. The purpose of this study is to encapsulate Janus nanoparticles (B) in a polymer nano-film (A) to obtain micro-nanocapsules by electrostatic layered self-assembly. The microcapsules break down under pressure, dissolution or melting to release B. The schematic diagram is shown in Figure 4.

### 2.7. Determination of Emulsified Resistance Coefficient 

Based on the single chemical agent or composite system of polymer and surfactant, both belong to non-Newtonian fluids. When the binary system and crude oil have not been emulsified, the macroscopic appearance of surfactant is pressure-lowering and drag-reducing [34,35,36]. When the binary system is emulsified with crude oil, the resistance coefficient of the system increases, and the morphology, rheological properties and particle size distribution of the system change after passing through porous media. In order to determine the percolation characteristics of the oil displacement system after emulsification, resistance coefficient and porous media were established to study the influence of resistance coefficient and porous media on the emulsifying ability of the binary system. Homogeneous cores with three oil-water ratios, three flow velocities, three surfactant concentrations and three permeabilities were selected for 27 groups of experiments. The resistance coefficients of five oil displacement systems are determined. The composition of five oil displacement systems is shown in Table 1.

**Figure 5:**
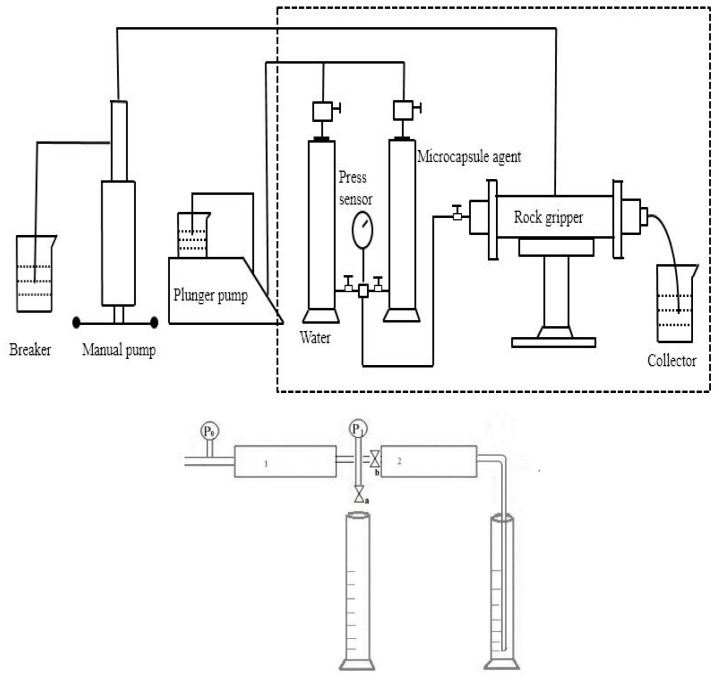
Diagram of series cores.

The experimental device is shown in Figure 5. The specific steps are as follows:

(1) The core is vacuum pumped for 4 h and saturated with brine. The porosity and permeability are calculated and the pressure P_W_ is recorded. 

(2) In each group of experiments, two sections of cores are connected in series. The former section is used to produce emulsion, and the latter section is used to study the seepage law of the emulsified system. The binary system of different schemes is injected into the first core to produce emulsion. The outlet is connected with a four-way valve. One end of the four-way valve is connected with sampling equipment, one end is connected with a pressure sensor, and the other end is connected with the core used in the experiment (the second core). 

(3) Sampling after the first core, observing whether the emulsion is generated by body microscope, real-time monitoring of the pressure curve of the second core, recording the final stable pressure P_E_, and measuring the shape and size change of the emulsion at the outlet. 

(4) To calculate the drag coefficient by selecting the average value of the increasing pressure of the injected emulsion, the resistance coefficient of R_f_ = P_W_/P_E_ is used to characterize the influence of different factors in porous media on the emulsifying capacity of the two-element system. Among them: P_W_—the pressure of core permeability measured by water, P_E_—the corresponding value of the stable section of the pressure curve after an injection of emulsion, and R_f_—the resistance coefficient of this emulsion. 

Reaction materials and conditions: Core parameters are shown in Table 2. The injection rate is 0.2 ml/min, the experimental temperature is 45 °C, and the water used in the experiment is tap water. The water permeability of the core used in the experiment is 10 × 10^−3^ μm^2^, 50 × 10^−3^ μm^2^ and 100 × 10^−3^ μm^2^, respectively.

Reaction materials and conditions: Core parameters are shown in Table 2. The experimental temperature is 45 °C, the water used for displacement is tap water, the saturated water for the core is 3700 mg/L formation salinized brine, and the saturated oil for the core is a simulated oil with an initial viscosity of 9.8 mPa·s.

27 groups of experimental parameters description: In order to analyze the influence of oil saturation, interfacial tension and displacement speed on the emulsification of oil displacement agent, three numerical values are selected for each influencing factor.

(1) The average value of the original oil saturation of the core is 70%, and the oil saturation after water flooding is about 50%. Therefore, in order to maintain the data gradient, the oil saturation of core is set to 70%, 50%, 30%. 

(2) Intelligent macromolecule nanocapsules with different interfacial tensions are selected because the degree of emulsification also depends upon the physicochemical properties of the surfactants. The concentration of surfactant was 0.05%, 0.1% and 0.2%, respectively, corresponding to three orders of magnitude of interfacial tension of 10^−1^ m N/m, 10^−2^ m N/m and 10^−3^ m N/m.

(3) In order to analyze the effect of shear rate on fluid properties, the shear resistance of oil displacement agents is different, based on different displacement rates. Therefore, 2 m/d,4 m/d and 8 m/d, are selected.

Description of displacement process: Take the core with 70% saturation, 0.2% mass concentration of smart polymer nanocapsules and 8 m/d flow rate as examples. The displacement agent is injected into the connected series cores, and the pressure changes at the entrance of the second core are observed, and the average value of the slower rise of the pressure is recorded. Then, according to the principle of controlling variables, one parameter experiment is changed at a time.

### 2.8. Adaptability Assessment

By measuring the particle size of the Janus Particle (JP) microcapsules at different temperatures, the function equation of particle size and temperature was established, and the temperature limit of these JPs microcapsules was determined. The effect of temperature on particle size can be expressed by the following formula (1):

(1) $$J=J_{0}*Exp(-GD/KT)*Exp(-G*/KT)$$

where J represents the assembly rate; $J_{0}$ represents the initial assembly rate; GD represents the change of diffusion activation free energy; G∗ represents the change of critical activation free energy; K represents the Boltzmann constant; T represents the reaction temperature.

**Figure 6:**
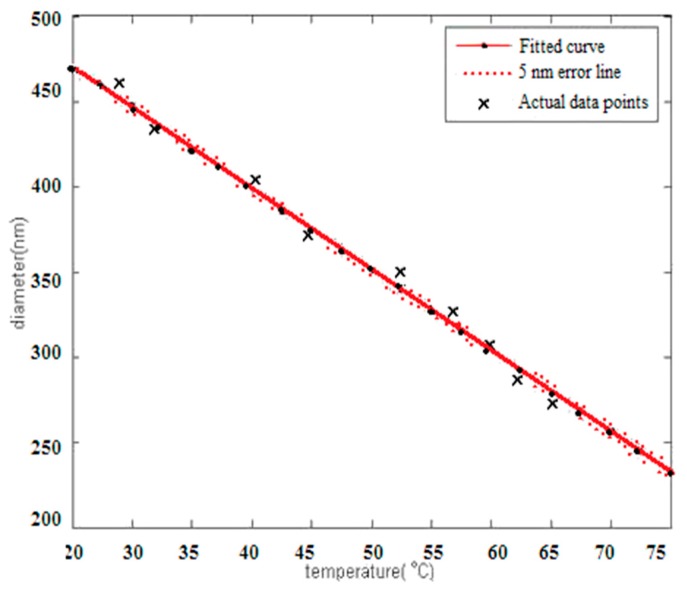
Fitting Curve of Particle Size and Temperature Change.

It can be seen from formula (1) that the assembly rate increases with the increase of reaction temperature, and the degradation or release rate is faster with the increase of temperature, while the release rate is faster than the assembly rate, and the final particle size decreases. The variation of particle size and temperature is shown in Figure 6.

Based on the proportional function of temperature and reservoir depth, the function relationship between particle diameter and temperature of oil displacement agent was indirectly verified by adjusting the temperature range from 20 to 75 °C. Therefore, the particle size can be controlled by adjusting the concentration of oil displacement agent and adapting the appropriate reservoir temperature.

### 2.9. Release Mechanism 

The degradation of microcapsules was tested in phosphate buffer solution at different temperatures. The release time and mechanism of polymer film on the outer wall and microcapsules inside the capsules were determined by degradation experiments. Based on the core-shell structure of the microcapsules, the polymer film on the outer wall of the capsules will degrade first, and the micro-nanoparticles in the capsules will release accordingly, while the micro-nanoparticles will theoretically deform due to the interfacial tension.

### 2.10. Reducing Displacement Pressure Experiment 

The experimental cores are all low permeability artificial homogeneous cores with a diameter of 2.5 cm. Five artificial homogeneous cores, C-1,2,3,4,5, with an average porosity of 16.3%, average water permeability of 25 × 10^−3^ μm^2^, and salinity of formation simulated brine of 7500 g/L, are selected. The displacement water is tap water. The experimental oil is the crude oil of the Daqing Oil Production Plant ( Daqing, in Heilongjiang province, People’s Republic of China). The experimental temperature is 45 °C. The pressure reduction experiments were carried out with five oil displacement agents of 0.4 wt% concentration. The results are shown in the Table 3. It can be seen from the table that after injection of oil displacement agent, the subsequent water displacement pressure decreases compared with that before injection. JANUS nanoparticle microcapsules have a significant effect on reducing displacement pressure, with a pressure reduction rate of 66.0%. The experimental results are shown in Table 3.

In order to clarify the phase change of the fluid in the core after the injection of smart polymer nanocapsules, two artificial homogeneous cores with the same physical parameters in series are used to carry out oil displacement experiments. The experimental cores are low permeability artificial homogeneous cores with a diameter of 2.5 cm, with a porosity of 16.3%, a pore volume of 7.8 cm^3^ and a water permeability of 25 × 10^−3^ μm^2^. Core 1 and core 2 were connected by four-way valves. The series core schematic diagram is shown in Figure 5.

**Figure 7:**
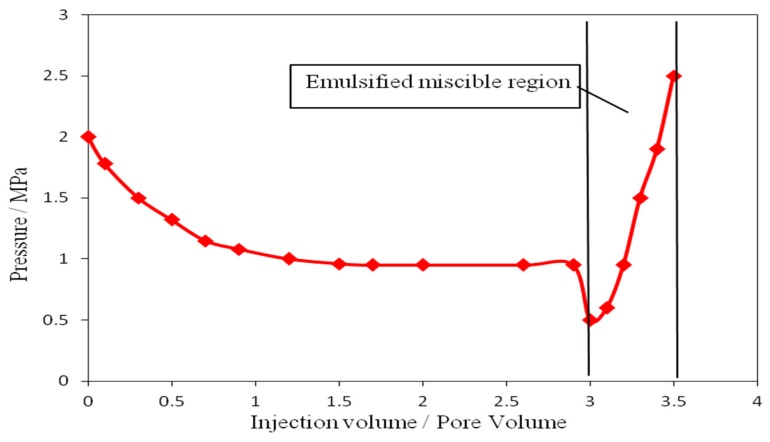
Curve of injection pressure versus injection volume.

Description of fluid process: Intelligent macromolecule nanocapsule dispersed aqueous solution was injected into core 1, and the four-way valve was opened to the valve a. Record the changes of pressure P_0_ and P_1_. When the pressure difference is stable, close the valve b and pay attention to the emulsification state of the produced liquid of the valve a. When the produced liquid is in the emulsified state, the valve b is then opened, the change of P_1_ is recorded, and the oil production at the outlet of core 2 is also recorded. The experimental results are shown in Figure 7.

Experiments show that the pressure drop of the smart polymer nanocapsules is obvious and then stabilizes with the increase of the injection volume. When the injection volume is 3 PV, the pressure first drops and then increases, indicating that the area is an emulsified miscible zone. This indicates that the wall of the smart polymer nanocapsules dissolves, and the Janus nanoparticles emulsify with crude oil to form an emulsion.

### 2.11. The Relationship between Oil Saturation of Core 2 and Time

Through water flooding-intelligent polymer nanocapsule flooding agent-subsequent water flooding experiments, the curve of recovery versus injection volume was obtained. The relationship between oil saturation and time is obtained based on core parameters and displacement experimental data according to recovery degree data. The formula is detailed in (2).

(2) Qoi·Vpt=(Qoi−QProduced liquid)·VpVp·PVv=Soit

$Q_{oi}$ represents the oil volume; $Q_{\mathrm{Produced}\;\mathrm{liquid}}$ represents the Produced oil volume;$S_{oi}$ represents the oil saturation;$V_{p}$ represents the pore volume, PV represents the injection volume, v represents the displacement speed; t represents the displacement time.

**Figure 8:**
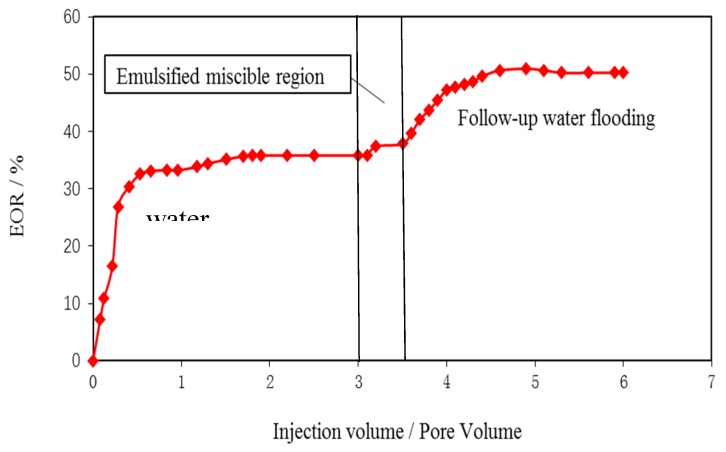
Relation Curve between Intelligent Polymer Flooding Agent Injection Rate and Recovery Rate.

**Figure 9:**
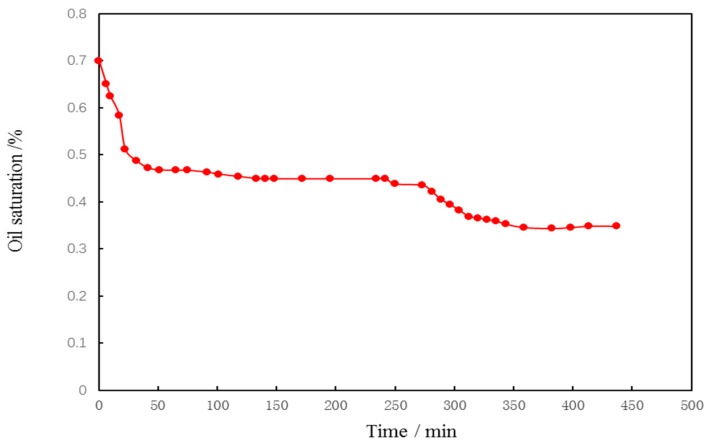
Chart oil saturation versus time.

The original oil saturation of the experimental cores is 70%. The curve of oil saturation with displacement time is obtained by conversion of the formula. Intelligent polymer flooding agent injection volume and recovery curve. The curve is shown in Figure 8, and the corresponding oil saturation curve with time is detailed in Figure 9.

## 3. Results

### 3.1. Characterization of Asymmetric Amphiphilic Nanoparticles 

#### 3.1.1. Fourier Transform Infrared Spectroscopy 

**Figure 10:**
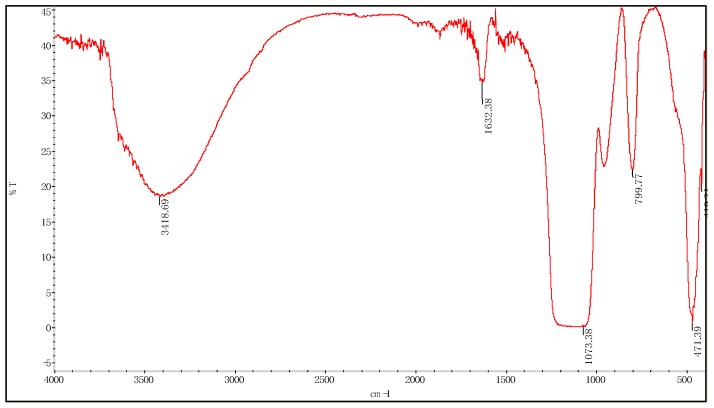
Fourier transform infrared spectroscopy of silica (bare) particles.

There are five characteristic absorption peaks in the infrared spectra of nano-silica prepared. The infrared spectra of prepared silica are shown in Figure 10. The broad peak of 3418.69 cm^−1^ is the anti-symmetrical stretching vibration peak of structural water-OH; the wide peak of 1632.38 cm^−1^ is the H-O-H bending vibration peak of structural water; the peak of 1073.38 cm^−1^ belongs to the bending vibration peak of Si-OH; the peak of 471.39 cm^−1^, and 419.31 cm^−1^ are the Si-O bending key vibration expansion peak. 

**Figure 11:**
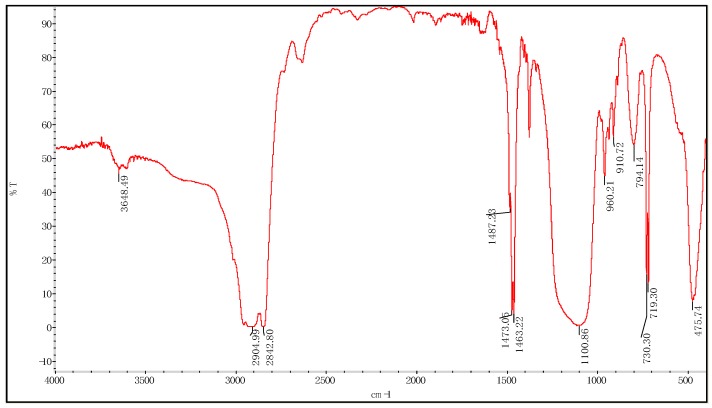
Fourier Transform Infrared Spectra of Silica (Emulsified) Particles.

The infrared spectra of the emulsified particles are shown in Figure 11. The infrared spectra of paraffin emulsified nano-silica showed 719.30 cm^−1^, 730.30 cm^−1^, 794.14 cm^−1^, 1463.22 cm^−1^, 1473.05 cm^−1^, 1487.23 cm^−1^, 2904.99 cm^−1^, 2842.82 cm^−1^, paraffin characteristic absorption peaks, 800-1250 cm^−1^ for C-C absorption peaks. Comparing with the infrared spectra of the emulsified particles and silicon dioxide, no new peaks appeared, which indicated that the emulsification of paraffin and silicon dioxide did not produce any chemical reaction, but only a physical combination.

**Figure 12:**
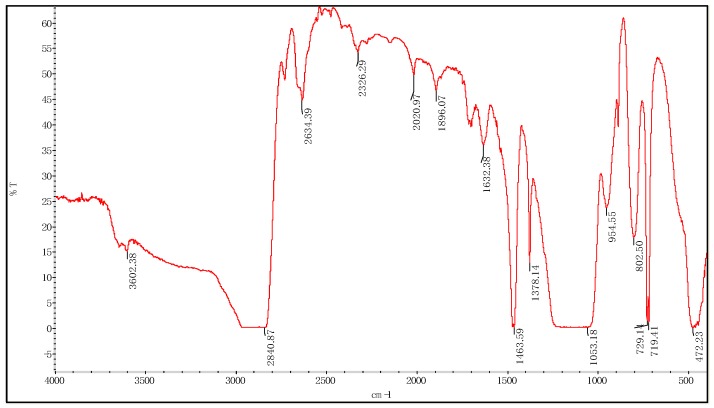
Fourier Transform Infrared Spectra of Silica (Modified) Particles.

The infrared image of the modified particles is shown in Figure 12. The general formula of the silane coupling agent is Y-R-SiX_3_, in which X is an alkoxy group capable of hydrolysis, Y represents an active group capable of reacting with polymers, and R is an organic carbon chain. When nano-silica is modified, the X group is hydrolyzed to silanol first, and then the hydroxyl groups on the surface of the nano-silica particles react to form chemical bonds, and these silane molecules are crosslinked to form T-type reticular membranes covering the surface of the nano-silica particles, so as to cover the surface of nano-silica by an organic layer. The vibration peaks of 2634.39 cm^−1^, 2326.29 cm^−1^, 2020.97 cm^−1^, 1896.07 cm^−1^-CH_3_/-CH_2_ were observed after modification with the KH570 coupling agent. The strong and wide characteristic absorption peak of Si-O-C was assigned to the band at 1053.18 cm^−1^, and the symmetrical stretching of Si-O-CH_3_ was assigned to 719.41 cm^−1^, 729.14 cm^−1^ and 802.50 cm^−1^, which indicated the existence of organic components on the surface of modified nano-silica. The copolymerization and condensation of organic and inorganic components between hydroxyl groups through covalent bonding indirectly prove the effective formation of functional JPs’ micelles.

#### 3.1.2. Diffraction Spectrum 

**Figure 13:**
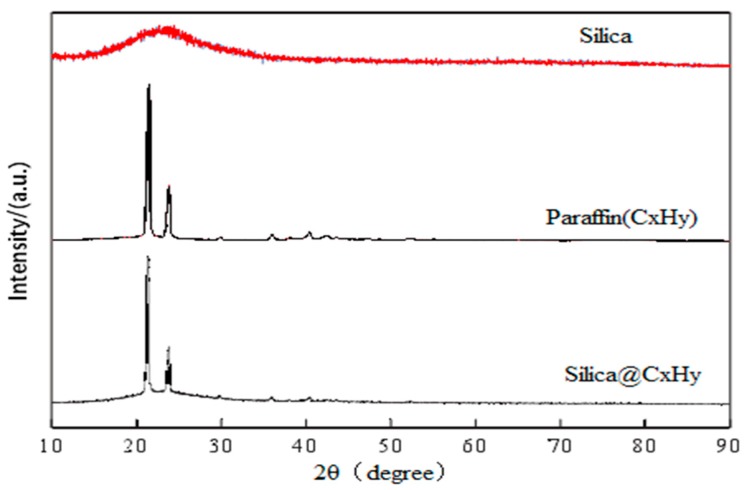
Comparison of Diffraction Spectra of Silica Nanoparticles, Paraffin, Silica@Paraffin.

The XRD (Diffraction Spectrum Contrast Diagram, Shimadzu Corporation, Osaka, Japan) of the sample is shown in Figure 13. The encapsulation of silicon dioxide after paraffin emulsification has no effect on the diffraction. Compared with the diffraction spectra of paraffin and silicon dioxide @ paraffin emulsified particles (silica particles emulsified by paraffin), the diffraction peaks of the emulsified particles are only a simple combination of the peaks of paraffin and silicon dioxide, and there are no new peaks, indicating that the combination of the two does not change the structure of the substance, but is only a physical combination, and the derivation of paraffin and emulsion particles. The peak position and half-peak width are identical, which indicates that the two structures are similar. At the same time, it confirms the conclusion of infrared spectroscopy.

#### 3.1.3. Laser Particle Size Distribution

**Figure 14:**
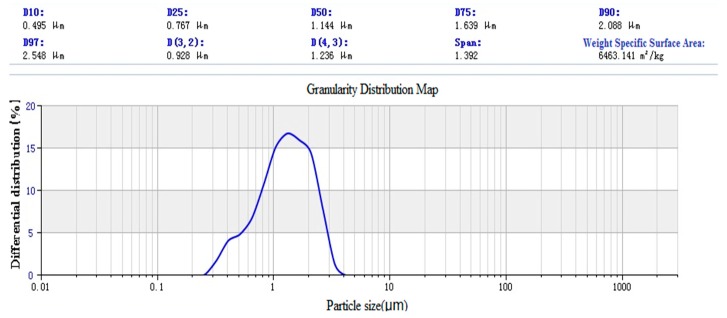
Laser particle size analysis of nano-silica.

**Figure 15:**
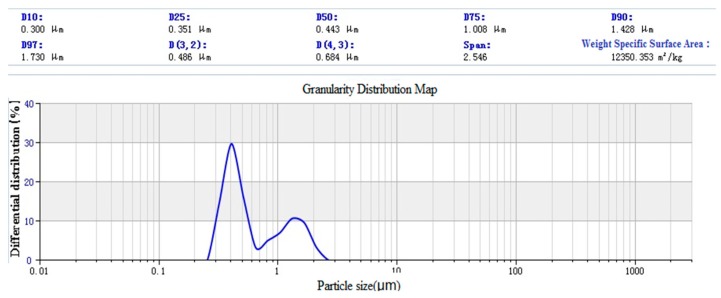
Paraffin @ Nano-SiO_2_ Laser Particle Size Analysis Diagram.

SiO_2_ was prepared by the Stöber method [7]. The size distribution of nano-SiO_2_ particles is shown in Figure 14. The results show that the average particle size of SiO_2_ particles is 1144 nm, and the specific surface area is 6463.141 m^3^/kg. The surface silicon hydroxyl density of SiO_2_ particles is 3.5 nm^2^. The paraffin @ nano-silica laser particle size analysis chart is shown in Figure 15. 

**Figure 16:**
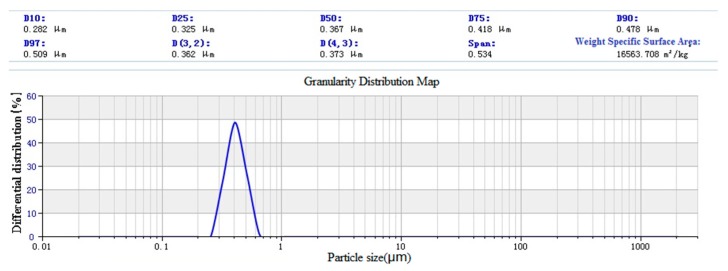
Laser particle size analysis of nano-silica modified by coupling agent.

After emulsification, the average particle size reduces to 443 nm, the specific surface area increases to 12350.353 m^3^/kg. It can be found that there are two peaks in the particle size distribution, and the main interference factor is identified as the cause of paraffin coating. During cooling, some particles are solidified by aggregated liquid paraffin, which leads to larger particle size in particle size measurement. A coupling agent modified nano-silica laser particle size analysis chart is shown in Figure 16. The average particle size after modification is 367 nm, and the specific surface area is 16563.708 m^3^/kg.

#### 3.1.4. Scanning Electron Microscope 

In the process of preparing JPs particles by the Pickering emulsion template method, nano silica particles are physically combined with paraffin dispersed particles, and at the oil-water interface, silica adheres to the surface of wax on the solidification stage, forming silica @ paraffin emulsified particles. 

**Figure 17:**
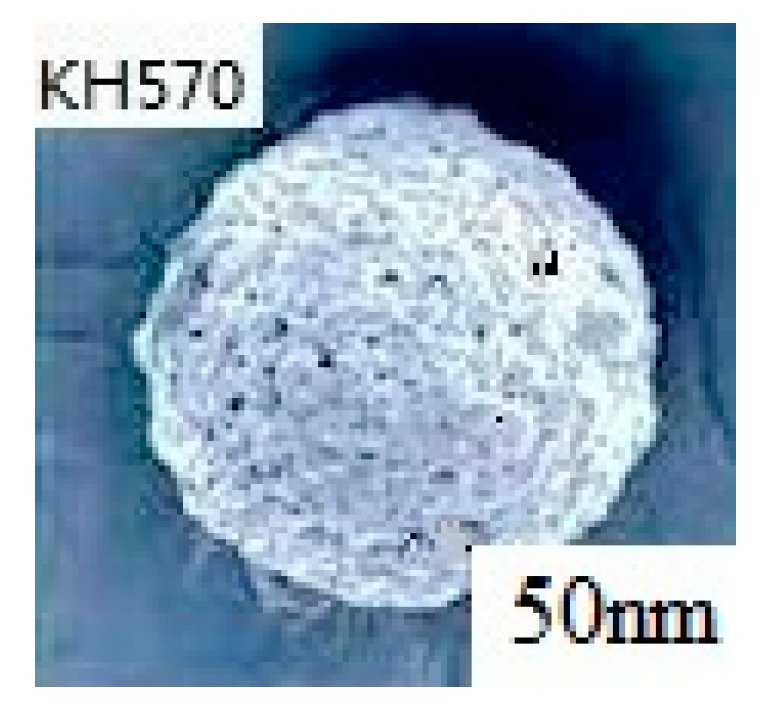
Scanning electron microscope of coupling agent modified nano-SiO_2_ Emulsified Rubber Particles.

The structure of emulsified rubber particles was analyzed by scanning electron microscopy. As shown in Figure 17, the stacking of KH570 modified silica on the surface of paraffin particles was homogenously packed. 

**Figure 18:**
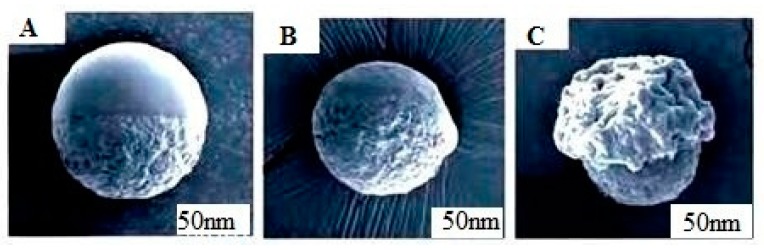
Electron Microscopic Scanning Image of Asymmetric Janus Nanoparticles Microcapsule Release. (**A**): at room temperature; (**B**): at 35 °C; (**C**): temperature exceeds 45 °C

The scanning electron microscopic images of JPs nanocapsules are shown in Figure 18. The morphology of JPs nanocapsules is shown in A at room temperature and B at 35 °C, the scanning electron microscopic images are shown in C when the dispersing temperature exceeds 45 °C.

**Figure 19:**
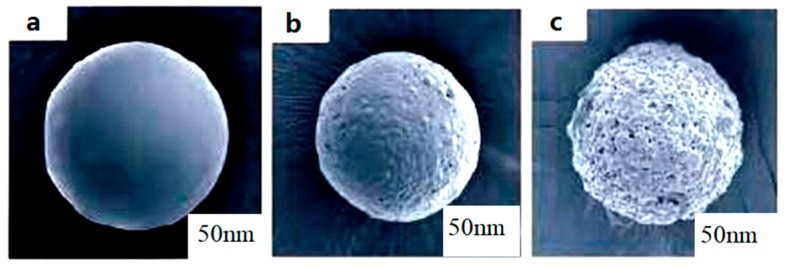
Electron Microscopic Scanning Image of Symmetrical Nanoparticles Microcapsule Release. (**a**): at room temperature; (**b**): at 35 °C; (**c**): temperature exceeds 45 °C

The scanning electron microscope images of symmetrical nanoparticles microcapsules are shown in Figure 19. The shape of microcapsules can be seen in a at room temperature, the dispersing temperature is b at 35 °C, and when the dispersing temperature exceeds 45 °C, the scanning electron microscope images are c.

Scanning electron microscopy (SEM) images of microcapsule release showed that the polymer shell gradually degraded to form a dispersed liquid system after increasing temperature, and the symmetrical/asymmetrical colloidal particles gradually detached from the shell at the same time of degradation. At the same time, the temperature response of the microcapsule was confirmed.

**Figure 20:**
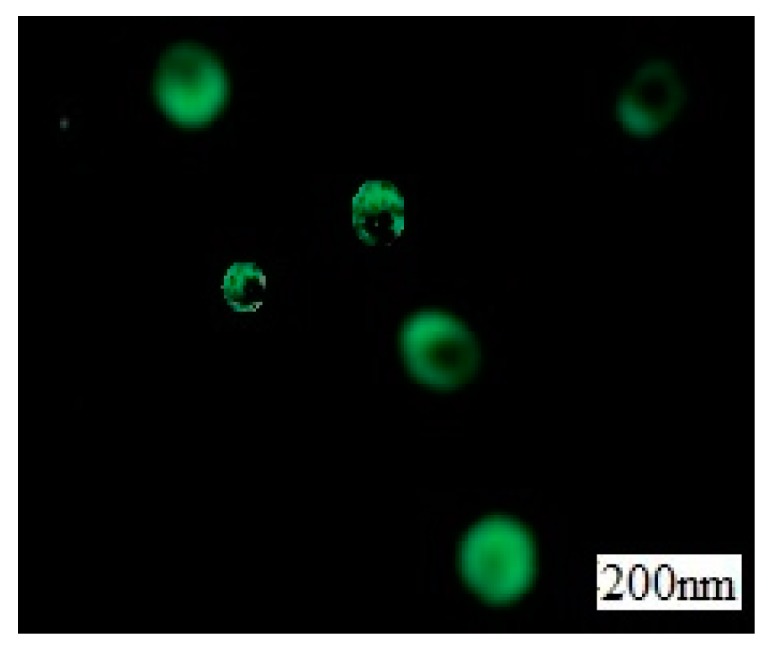
Schematic diagram of fluorescence microscope of amphiphilic Janus nanoparticles.

The fluorescence microscopic photographs of amphiphilic Janus nanoparticles are shown in Figure 20. Observations show that Janus nanoparticles are successfully labeled by FICT. 

### 3.2. Interface Properties of Amphiphilic Nanoparticles 

In order to study the interfacial properties of amphiphilic nanoparticle dispersions, the evolution of surface tension and interfacial tension with time at different concentrations of amphiphilic nanoparticles was measured. The surface tension of liquid was measured by the slice method. The critical micelle concentration is determined by surface tension method. Based on the fact that the surface tension of solution depends on the concentration of surfactant monomer, the concentration of monomer almost remains unchanged after micelle formation. Therefore, there will be a significant inflection point when the surface tension changes with the concentration. The inflection point is the critical micelle concentration (CMC).

**Figure 21:**
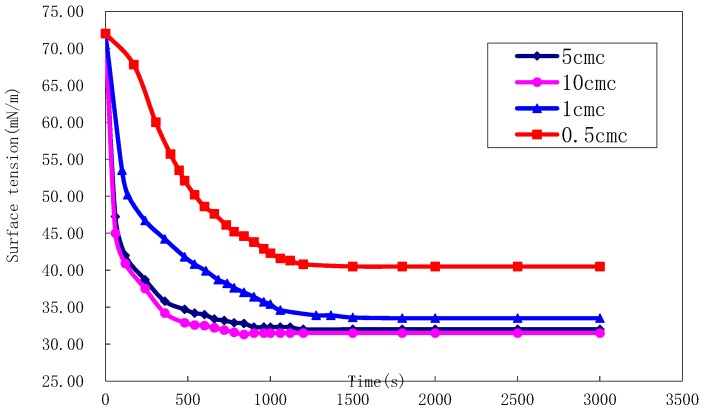
Surface tension of different critical micelle concentration of nanophilic particles.

**Figure 22:**
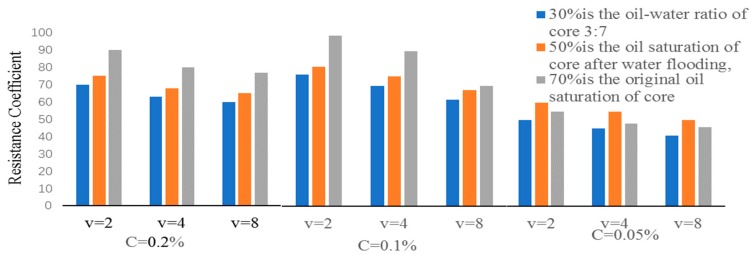
Cross-sectional analysis of 27 groups of emulsified resistance coefficient histograms.

As shown in Figure 22, the surface tension decreased rapidly in the early stage, and then reached equilibrium at a different concentration. As the concentration of amphiphilic nanoparticles increases, the equilibrium surface tension decreases until the critical concentration is reached. At this time, the adsorption of amphiphilic nanoparticles at the air-water interface reaches saturation, and the equilibrium surface tension drops to about 31 mN/m. It can be found from the Figure 21 that the surfactant concentration is more than double CMC, the surface tension basically does not change significantly.

The interfacial tension of liquid was measured by the suspension drop method. As shown in Table 4, the measurement range of ultra-low interfacial tension is 10^−2^ to10^−4^ m N/m, the spindle speed range is 1000 to 9000 rpm, and the temperature range is from room temperature to 190 °C. A simulated crude oil with a viscosity of 9.8 mPa·s was used. 

The critical micelle concentration of surfactant was obtained at 45 °C and 2000 rpm. The interfacial tension between oil and water reached 10^−4^ m N/m.

### 3.3. Emulsifying Ability 

The emulsification index test was carried out at room temperature and 45 °C. The emulsification index is the ratio of emulsification volume to total volume after 24 h to 72 h. The higher the emulsification index, the better the emulsification effect and the stable emulsification performance. Comparing the volume of emulsifying layer with the total volume at a certain time can also show the emulsifying ability. As shown in Table 5, it can be concluded that the volume of the emulsified layer does not increase with the increase of surfactant concentration, but decreases with the increase of surfactant concentration after reaching a certain volume. 

### 3.4. Determination of Emulsified Resistance Coefficient of Amphiphilic Janus Nanoparticles System 

Based on the droplet collision and coalescence, the interfacial free energy of the system decreases, so the emulsification process is a thermodynamically unstable system. When the Janus particles are self-assembled at liquid-liquid interface, the stabilization energy is three times that of the homogeneous particles. This indicates that Janus particles have extremely high interfacial activity, and can be used as efficient surfactant to stabilize the emulsion. Therefore, the emulsified resistance coefficient of this JPS system was determined. Considering three main factors, 27 groups of orthogonal experiments were carried out. The range of main parameters is detailed in Table 6. 

The concentration of surfactant was 0.05%, 0.1% and 0.2%, respectively, corresponding to three orders of magnitude of interfacial tension of 10^−1^ m N/m, 10^−2^ m N/m and 10^−3^ m N/m. As shown in Figure 22, when the concentration of surfactant is 0.1% and 0.2%, the drag coefficient is relatively large, and the value is close. It can be concluded that the emulsified state of the system with concentration greater than 0.1% is relatively stable. Increasing the concentration of surfactant cannot improve the blocking ability of the system migration. 

The increase of oil saturation and drag coefficient of the system are mainly due to the change of the emulsified form of the emulsion. With the increase of oil saturation, the emulsified state of the system is changed from O/W to W/O, which improves the fluidity ratio and presents the phenomenon of pressure rise resistance coefficient increasing. Optical microscopy was used to observe the emulsification morphology at the core outlet. It was found that only 70% of the saturation formed W/O type, 30% and 50% of the oil saturation were O/W type. 

The larger the flow velocity is, the smaller the drag coefficient is. Both binary and emulsified systems are non-Newtonian fluids with shear thinning properties. At high capillary number, the retained emulsion droplets can pass through the fine pore throat, the particle size decreases and the pressure decreases. It shows that the resistance coefficient is slightly smaller than that of the binary system, but because of high-speed shearing, the emulsifying ability of the system will be enhanced. Different emulsified systems and binary systems will appear shear thickening, and the resistance coefficient will increase slightly. The emulsified state of polymeric nanoparticle oil displacement agent composite system is pseudoplastic fluid. Under experimental conditions, shear thickening phenomenon is found, and then the resistance coefficient will increase slight.

In the experiment, the dilution method was used to observe the change of the emulsified state of the produced liquid. If it can be miscible, its continuous phase must be water phase, so it is O/W type, if not W/O type. After injecting 0.85 times of pore volume (PV) into the conventional surfactant binary system, the system changed from emulsified state to oil-water separation state, and the emulsified state recovered after 3 PV. In the displacement process, smart polymer nanocapsules enter the emulsified state at 3 PV, which indicates that there is almost no adsorption loss on the surface of smart polymer nanocapsules and rocks at the initial stage of injection. With the increase of injection volume, the emulsified state did not break down and was relatively more stable.

## 4. Discussion

### 4.1. Preparation of Amphiphilic Asymmetric Nanoparticles 

In the process of synthesizing Janus particles by the Pickering emulsion method, the amphiphilic Janus nanoparticles were obtained by increasing the CTAB concentration of cationic surfactant and assisting the migration of silica particles to the oil-water interface. The functional groups (Si-OH) were modified by silane coupling agent to increase the surface roughness of silica particles. The wetting reaction is the common result of the functional group transformation on the surface of the particles, and the change of the surface roughness of the modified silicon particles. 

With the bare silica particles (E = 72 MJ/m^2^) compared with silica particles modified by silane coupling agent showed lower surface free energy values (E = 45 MJ/m^2^). The smaller the surface energy, the stronger the lipophilicity. Therefore, Janus nanoparticles are hydrophobic and hydrophobic compared with silica particles. The asymmetric surface modification of Janus particles was confirmed by fluorescence microscopy, and the active surface of silicon dioxide particles, which had been shielded by wax after chemical modification, was also confirmed. 

### 4.2. Interfacial Activity and Emulsifying Ability of Amphiphilic Asymmetric Nanoparticles 

The concentration of amphiphilic nanoparticles satisfies the Gibbs stability criterion and is significantly lower than that of homogeneous nanoparticles. The two nanoparticles with high affinity show a negative free energy formed by electrostatic interactions. The binary system of conventional surfactant changed into oil-water separation state with the increase of injection amount, and then the emulsified state recovered. However, the amphiphilic asymmetric nanoparticles had little adsorption loss with the rock surface, and the emulsified state did not break down during the increase of injection amount, which was relatively more stable.

## 5. Conclusions

The purpose of this study was to assemble polymeric nanocapsules using modified asymmetric amphiphilic nanoparticles as surfactants. These particles exhibit remarkable interfacial activity at the oil-water interface, and the surface activity of these nanoparticles is better than that of conventional surfactants. When the interface reaches saturated adsorption, with the increase of particle concentration, the equilibrium surface tension decreases to below 31 mN/m, and the interfacial tension decreases to 10^−4^ mN/m. In addition, the relationship between particle interface assembly and polymer capsule wall was studied. After meeting the Gibbs stability criterion, the intelligent micro-nano capsule flooding system has a better plugging ability. It is noteworthy that amphiphilic nanoparticles have the potential to be used as colloidal surfactants to enhance oil recovery due to their unique anisotropic structure.

## Figures and Tables

**Table 1:** Composition of five oil displacement systems.

Oil Displacement System	Composition (w t%)
Surfactant (sodium heavy alkylbenzene sulfonate)	0.3
Uniform Modified Nanoparticles	0.3
Asymmetric Modified Nanoparticles	0.3
Uniform modified nanoparticle microcapsules	0.3
Asymmetric amphiphilic nanoparticles microcapsules	0.3

**Table 2:** Core parameters.

Parameters	Core Type	
	Low Permeability Core L1	Low Permeability Core L2
Core length (cm) Core diameter(cm)	9.80	9.81
	2.5	2.5
Water permeability measurements(10^−3^μm^2^)	51.6	53.1
Pore volume(cm^3^)	6.3	6.5
Porosity (%)	13.1	13.5

**Table 3:** Laboratory experimental results on reducing displacement pressure of surface core.

Oil Displacement Agent	Pore Volume Multiple of Water Flooding	Pressure at the End of Water Flooding (M·Pa)	Follow-Up Water Drive Pressure after Displacement by Oil Displacement Agent (M·Pa)	Pressure Reduction (%)
Sodium Heavy Alkylbenzene Sulfonate	3.76	0.53	0.27	49.1
Uniform Modified Nanoparticles	2.87	0.53	0.23	56.6
Asymmetric Modified Nanoparticles	2.98	0.53	0.21	60.4
Uniform modified nanoparticle microcapsules	3.18	0.53	0.21	60.4
JANUS Nanoparticle microcapsules	3.26	0.53	0.18	66.0

**Table 4:** measuring of Surface/Interfacial tension.

JPs (wt%)	Surface Tension (m N/m)	Interfacial Tension (m N/m)
0	72.0	10
0.05	45.3	1.5 × 10^−1^
0.1	40.2	5.1 × 10^−2^
0.2	37.9	8.4 × 10^−3^
0.4	33.5	2.3 × 10^−3^
0.8	31.0	3.3 × 10^−4^

**Table 5:** Emulsifying performance measurement.

**Sodium Heavy Alkylbenzene Sulfonate (mol/mL)**	**Volume of Emulsified Layer (mL)**			
	**0 h**	**24 h**	**48 h**	**72 h**
0.4	5.5	5.5	5.0	5.0
0.6	7.0	7.0	6.5	6.0
0.8	8.0	8.0	8.0	7.5
1	7.0	6.5	6.0	5.0
1.2	5.5	5.0	3.5	3.0
**Modified nanoparticles (mol/mL)**	**Volume of emulsified layer (mL)**			
	**0 h**	**24 h**	**48 h**	**72 h**
0.4	6.5	6.5	6.5	6.5
0.6	7.0	7.0	7.0	7.0
0.8	9.0	9.0	9.0	9.0
1	6.0	6.0	6.0	6.0
1.2	5.5	5.0	4.5	4.5
**Asymmetric** **Modified nanoparticles (mol/mL)**	**Volume of emulsified layer (mL)**			
	**0 h**	**24 h**	**48 h**	**72 h**
0.4	7.5	7.5	7.5	7.5
0.6	8.0	8.0	8.0	8.0
0.8	9.0	9.0	9.0	9.0
1	7.0	7.0	7.0	7.0
1.2	6.5	6.0	6.0	5.0

**Table 6:** Orthogonal test parameters of drag coefficient.

Considerations	Range of Values		
oil saturation (%)	30	50	70
Surfactant Concentration (%) <interfacial tension (mN/m)>	0.05 (1.5 × 10^−1^)	0.1 (5.1 × 10^−2^)	0.2 (8.4 × 10^−3^)
Velocity of flow (m/d)	2	4	8
